# Can hybrid hyaluronic acid represent a valid approach to treat rizoarthrosis? A retrospective comparative study

**DOI:** 10.1186/s12891-017-1809-5

**Published:** 2017-11-13

**Authors:** Sara Tenti, Nicola Antonio Pascarelli, Stefano Giannotti, Mauro Galeazzi, Nicola Giordano, Antonella Fioravanti

**Affiliations:** 1 Rheumatology Unit, Department of Medicine, Surgery and Neurosciences, University of Siena, Viale Bracci 1, 53100 Siena, Italy; 2 Section of Orthopedics and Traumatology, Department of Medicine, Surgery and Neurosciences, University of Siena, Siena, Italy; 3 Scleroderma Unit, Department of Medicine, Surgery and Neurosciences, University of Siena, Siena, Italy

**Keywords:** Trapeziometacarpal joint, Intra-articular therapy, Hybrid hyaluronic acid, Retrospective comparative study

## Abstract

**Background:**

Osteoarthritis (OA) of the trapeziometacarpal joint (TMJ) is a disabling condition with a significant impact on quality of life. The optimal management of hand OA requires a combination of non-pharmacological and pharmacological treatments that include intra-articular (i.a.) therapy. EULAR experts recommend corticosteroid injections in TMJ OA and underline the usefulness of hyaluronic acid (HA). The aim of this study was the assessment of the efficacy and tolerability of i.a. injections of a hybrid formulation of HA (Sinovial H-L®) in comparison to triamcinolone in patients with TMJ OA.

**Methods:**

This 6-months observational comparative study, retrospective analyzed the medical records of 100 patients with monolateral or bilateral TMJ OA, treated with two injections of Sinovial H-L® (Sinovial H-L Group) or of triamcinolone acetonide (Triamcinolone Group). Clinical assessments were recorded at the time of the first and second injection and after one, 3 and 6 months.

The primary outcomes were the change in global pain on a Visual Analogue Scale (VAS) and in hand function evaluated by the Functional Index for Hand OA (FIHOA) from baseline to month 6. Secondary outcomes were the improvement of the duration of morning stiffness, Health Assessment Questionnaire (HAQ) and the Medical Outcomes Study 36-Item Short Form (SF-36). The comparison between the two groups of treatment were performed with the Wilcoxon rank-sum test for continuous variables and with chi-square or Fisher exact test for categorical variables. Statistical significance was set at *p* < 0.05.

**Results:**

Both therapies provided effective pain relief and joint function improvement, but the benefits achieved were statistically significantly superior in the Sinovial H-L Group than the Triamcinolone Group after one month (*p* < 0.01) from the beginning of the therapy and during the 6-months follow-up (*p* < 0.001). Furthermore, Sinovial H-L® was associated with a significant decrease in the duration of morning stiffness and with a significant improvement in the HAQ score and physical component summary (PCS)-SF-36.

**Conclusions:**

Our results suggested that the hybrid formulation of HA may be more effective than triamcinolone in pain relief and joint function improvement with a rapid and persistent effect, resulting a valid alternative to steroid in the management of TMJ OA.

**Trial registration:**

ClinicalTrials.gov, date of registration: June 14, 2017, NCT03200886. The present trial was retrospectively registered.

## Background

Osteoarthritis (OA) of the trapeziometacarpal joint (TMJ), also called rhizarthrosis, is a common disease, mostly affecting post-menopausal women; its prevalence is 30% among women over the age of 65 reaching a peak of 91% in patients older than 80 years [1]. This condition can evolve painless or determines pain, swelling and deformities with a significant impact on the quality of life [2]. Severity of TMJ OA was classified radiologically using either Kellgren-Lawrence I-IV or Eaton and Glickel I-IV scale [3, 4]. Despite its high prevalence and disability, the therapeutic options in TMJ OA are still limited and few have been investigated. According to the European League Against Rheumatism (EULAR), the optimal management of hand OA requires a combination of non-pharmacological (such as local application of heat, exercise, ultrasound and splints for thumb base OA) and pharmacological treatments [5]. The efficacy of intra-articular (i.a.) therapy is still the subject of debate. EULAR experts recommend corticosteroid injections in TMJ OA and state that “hyaluronic acid (HA) may be useful” [5]; on the contrary, both of these modalities are not supported by the American College of Rheumatology (ACR) [6]. More recently, in the consensus on viscosupplementation for the management of OA, HA therapy was recommended as a second-line treatment after failure of non-pharmacological modalities, only in early stages of the disease [7]. Corticosteroid injections are a mainstay of therapy in OA, especially for patients with pain refractory to oral treatments, although their use is limited by their potential adverse effects [8, 9]. Into the TMJ, steroid injections can lead to significant short-term benefits, such as pain relief and improved function, particularly from 1 to 3 months post-administration [10]. HA, that belongs to the glycosaminoglycan family, is the main component of the cartilage matrix in normal joints and it’s responsible of lubrification, shock absorption and visco-elastic properties of synovial fluid (SF). Viscosupplementation is nowadays accepted as a safe and effective treatment of knee OA and its uselfulness is recently recognized also for other joints, such as hip, ankle, shoulder, temporomandibolar joint and TMJ [7]. The literature about the use of i.a. HA for rhizarthrosis is limited, although some studies have shown promising results, in particular in increasing functional capacity [11].

Different HA formulations are currently available worldwide and their properties depend on the concentration and molecular weight (MW); on this basis, we can distinguish low (range:500–730 kDa), intermediate (800–2000 kDa) and high (average: 6000 kDa) MW preparations [12]. Furthermore, a hybrid HA formulation, Sinovial High-Low (H-L)® was recently developed and approved by European Medicines Agency (EMA) for the treatment of OA; it is featured by a bimodal MW profile distribution with high and low MW fractions combined. Sinovial H-L® presents the same rheological properties of a high MW HA and thanks to the low MW fraction is able to stimulate the anabolic activity of OA chondrocytes, in vitro [13, 14].

The aim of the present observational, retrospective, comparative, study was the assessment of the efficacy and tolerability of i.a. injections of Sinovial H-L® in comparison to triamcinolone in patients with TMJ OA.

## Methods

### Participants

This is an observational study with retrospective review of medical records which was conducted in the Rheumatology Unit of the Azienda Ospedaliera Universitaria Senese (Italy), after obtaining the permission by the local committee of Azienda Ospedaliera Senese (decision no. COMB-IAL-V-TRIAM 01, 22th May 2017) and registered on http://www.clinicaltrials.gov (NCT03200886). We analyzed the records collected in the departmental archives of outpatients affected by monolateral or bilateral TMJ OA, according to the ACR criteria for hand OA [15], and who were treated with i.a. Sinovial H-L® or triamcinolone acetonide from December 1st, 2015 to December 1st 2016. In case of bilateral TMJ OA, we defined the target hand as the patient’s most symptomatic hand or, when both hands were equally painful, the patient’s dominant hand.

We included in our analysis the records of patients of both sexes, aged between 45 and 75 years who had clinical symptoms of TMJ OA for at least 3 months and defined as global hand pain score superior to 30 mm on a 0–100 Visual Analogue Scale (VAS) and a Functional Index for Hand OA (FIHOA) score of at least 6.

Furthermore, to be included in the study, the patients have had a radiographic evidence of TMJ OA within the previous 6 months with a radiological score of II-III (using the Kellgren method) [3].

Patients with a history of any inflammatory joint disease, septic arthritis, major trauma or prior surgery of the hand, wrist, and elbow, coagulation disorders, severe comorbidity and those who underwent therapy with chondroitin sulfate, glucosamine, diacerein, steroids by any route of administration and i.a. injection of any joint with corticosteroids or HA during the previous 6 months were excluded. All patients were fully informed of the characteristics of the study and gave written informed consent for the collection and publication of anonymous data.

### Interventions

All patients were treated according to our routinary care and EULAR recommendations for the management of hand OA [5]. We have standardized patient selection by only including patients who had received specific HA and steroid concentration or formulation.

The choice of the preparation to be injected is influenced by the cost of HA that is not covered by our National Health Service (NHS), opposite to steroids. Furthermore, a known history of allergy to HA, precludes the use of this treatment.

The patients treated with HA have received one cycle of two injections (at baseline and 15 days apart) of 1 ml of Sinovial H-L® (3.2% - 16 mg + 16 mg, Ibsa) (Sinovial H-L Group) and the others have received two i.a. injections (at baseline and 15 days apart) of 0.5 ml of triamcinolone acetonide (Kenacort® 20 mg, Bristol-Myers Squibb Srl) (Triamcinolone Group). Triamcinolone acetonide is the injectable corticosteroid usually prescribed in our routine care.

Sinovial H-L® is an hybrid form of HA obtained through thermo-chemical processes from the combination of high (1100–1400 kDa) and low (80–100 kDa) MW fractions.

According to our routinary care, to receive the therapy, patients sat with the target hand in a semi-prone position on a table. After palpating the TMC joint space, laterally to the abductor pollicis longus tendon, within the anatomic snuffbox, the joint was injected with a 22-gauge needle after skin cleansing with 10% povidone iodine and then with ethyl chloride.

### Outcomes

The patient’s assessment of global hand pain on a 0–100 mm VAS with 0 representing the absence of pain and the FIHOA score validated in Italian language [16, 17] were routinely recorded and documented in our centre at the time of the first injection and of the second i.a. administration (two weeks), and after one, 3 and 6 months.

The primary outcome criteria of our study were the change of VAS and FIHOA from baseline to month 6.

The FIHOA score represents a quantitative measure of functional disability of the hands; it contains 10 items and is an investigator-administered questionnaire. Patients are asked to answer each item using a four-point Likert scale: 0 = possible without difficulty, 1 = possible with slight difficulty, 2 = possible with considerable difficulty, 3 = impossible. The range of scores is 0–30 and the highest values indicate the worst functionality [17, 18].

Furthermore, we chose as secondary outcomes the change of the duration of morning stiffness, the Italian version of the Health Assessment Questionnaire (HAQ) and the validated Italian version of the Medical Outcomes Study 36-Item Short Form (SF-36) from baseline to month 6.

HAQ is a self administered questionnaire developed to measure disability consisting of 8 sections: dressing, arising, eating, walking, hygiene, reach, grip, and activities and ranging from 0 to 3 with a higher score corresponding to worse disability [19, 20].

SF-36 is a widely used measure of health and well-being, including two main domains, mental and physical component summary (MCS and PCS respectively), that investigates 8 different areas of perceived health, such as physical functioning, physical role, bodily pain, general health, vitality, social functioning, emotional role and mental health. Scores range from “0 to 100” where “0” indicates the worst condition and “100” indicates the best possible condition [21, 22].

All adverse events, whether reported spontaneously by the patients or observed by the physician, were recorded, describing the severity and any possible correlations with the treatment.

### Statistical analysis

A sample size of 41 patients in each group was required to demonstrate a difference in change pain scores between the Sinovial H-L® and Triamcinolone Group of 10 mm (on a 0-100 mm VAS scale) at 6-months follow-up, assuming a standard deviation (SD) of 15 mm, with a power of 80% and an alpha error of 0.05.

The chi square test, t test or Kruskal-Wallis test, as appropriated, were used to evaluate the differences in the clinical and demographical data between groups at basal time.

Data are expressed as mean and SD for continuous variables. Categorical variables are expressed as frequency and percentages. The comparison between the two groups of treatment were performed with the Wilcoxon rank-sum test (Mann-Whitney test), for continuous variables, and with chi-square or Fisher exact test for categorical variables. Statistical significance was set at *p* < 0.05.The software Stata 13 was used for the analysis.

## Results

**Fig. 1:**
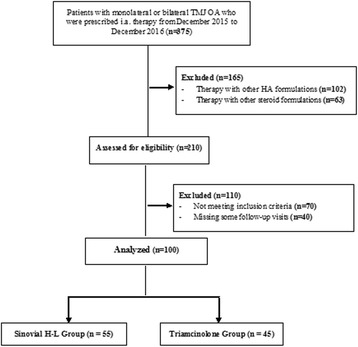
Flow diagram of the study population. OA: Osteoarthritis; TMJ: trapeziometacarpal joint; i.a.: intra-articular; HA: Hyaluronic acid

Within the period of one year between December 2015 and December 2016 i.a. therapy for monolateral or bilateral TMJ OA with HA or steroid was prescribed to 375 patients. 165 patients were excluded from our analysis because they were treated with different formulations of HA or steroid. Of the 210 remaining patients, 70 didn’t meet the inclusion criteria and 40 missed some follow-up visits. Finally, a total of 100 patients were selected according to the inclusion and exclusion criteria and further analyzed: 55 in the Sinovial H-L Group and 45 in the Triamcinolone acetonide Group (Fig. 1).

Baseline demographic and clinical characteristics of the study patients are summarized in Table 1. No significant differences were observed between the two groups about demographical and clinical data except for the percentage of patients who used to smoke or with diabetes and for the serum levels of total and LDL cholesterol; the study population was well representative of the hand OA population: mostly women (69%), mean age about 67 years with Kellgren and Lawrence radiological grades II and III equally distributed. For what concerns the outcome measures, no significant differences were registered between the two groups except for SF-36 MCS.

**Table 1:** Demographic and clinical characteristics of the study patients

	Sinovial H-L Group	Triamcinolone Group	*p* value
	(*N* = 55)	(*N* = 45)	
Age (years)	68.6 ± 9.4	65.5 ± 9.9	0.127
Sex, no. male/female	20/35	12/33	0.261
% with dominant right hand	100	100	1.000
BMI (kg/m^2^)	24.4 ± 4.0	24.4 ± 3.8	0.761
Disease duration (months)	88.65 ± 68.21	92.7 ± 62.7	0.179
Radiographic Score (K-L grade) no. (%)			
II	30 (54)	25 (56)	0.963
III	25 (46)	20 (44)	0.851
Marital status, no. (%)			
Unmarried	4 (7.2)	4 (8.8)	
Married	42 (76.5)	36 (80.0)	
Widow	5 (9.1)	2 (4.4)	
Divorced	4 (7.2)	3 (6.8)	0.628
Education, no. (%)			
None	2 (3.6)	3 (6.6)	
Primary	12 (21.8)	6 (13.3)	
Middle	15 (27.27)	8 (17.7)	
High	19 (34.54)	25 (55.8)	
University	7 (12.79)	3 (6.6)	0.383
No. (%) of smokers	1 (1.81)	6 (13.3)	**0.035**
No. (%) of patients with diabetes	1 (1.81)	8 (17.7)	**0.009**
No. (%) of patients with cardiovascular disease	3 (5.4)	4 (8.8)	0.598
No. (%) of patients with hypertension	22 (40.0)	20 (44.4)	1.000
No. (%) of patients with tyroid disease	8 (14.5)	10 (22.2)	0.447
Total Cholesterol (mg/dL)	220.2 ± 36.3	208.8 ± 39.1	**0.006**
LDL Cholesterol (mg/dL)	143.2 ± 30.5	134.2 ± 28.1	**0.004**
HDL Cholesterol (mg/dL)	63.5 ± 17.2	64.3 ± 18.0	0.781
Triglycerides (mg/dL)	139.7 ± 47.6	137.7 ± 50.1	0.410
ESR (mm/h)	16.2 ± 11.6	18.3 ± 12.2	0.081
CRP (mg/dL)	0.19 ± 0.29	0.3 ± 0.33	0.094
Glycemia (mg/dL)	94.7 ± 14.1	96.9 ± 17.9	0.371
VAS Pain (mm) (0–100)	58.5 ± 16.2	61.8 ± 17.6	0.320
VAS Stiffness (min)	9.7 ± 11.8	8.9 ± 4.1	0.185
FIHOA (0–30)	12.2 ± 4.3	12.9 ± 4.2	0.485
SF-36 PCS	35.9 ± 9.5	38.3 ± 8.7	0.191
SF-36 MCS	50.4 ± 7.7	41.4 ± 9.5	**0.001**
HAQ (0–3)	0.82 ± 0.44	0.88 ± 0.47	0.704

Data are expressed as mean ± SD

*BMI* Body mass index (body weight divided by the square of the height), *K-L* grade Kellgren Lawrence grade, *HDL-C* High-density lipoprotein cholesterol, *LDL-C* Low-density lipoprotein cholesterol, *ESR* Erythrocyte sedimentation rate, *CRP* Serum C-reactive protein, *VAS* Visual Analogue Scale, *FIHOA* Functional Index for Hand Osteoarthritis, *SF-36 PCS* Physical Components Summary, *SF-36 MCS* Mental Component Summary, *HAQ* Health Assessment Questionnaire

*p* value expressing a significant difference between groups are in bold style

**Fig. 2:**
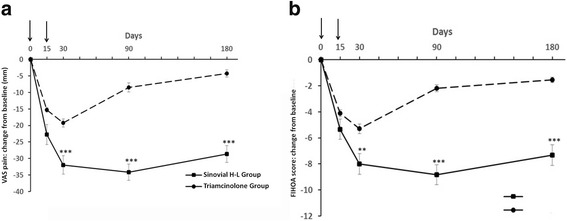
**a, b** Changes from baseline at each evaluation time point for the patient’s assessment of global hand pain by Visual Analogue Score (VAS, 0–100 mm) (**a**) and Functional Index for Hand Osteoarthritis (FIHOA) score (**b**) in the study groups. The arrows indicate the i.a. injections **p < 0.01; ***p < 0.001 (Wilcoxon rank-sum test) Sinovial H-L Group vs Triamcinolone Group

Improvement in the patient’s assessment of global hand pain was significantly more pronounced in the Sinovial H-L Group than in the Triamcinolone Group (mean ± SD −28.66 ± 16.92 versus −4.25 ± 6.85 mm; between-group difference in the amount of change 24.41 mm [*p* < 0.001]). The intergroup difference in absolute global pain levels at 6 months was −27.29 mm in favor of the Sinovial H-L Group (29.86 ± 20.13 versus 57.15 ± 14.46 in the Triamcinolone Group). The difference between groups became significant (*p* < 0.001) after one month from the beginning of the therapy and persisted until 6 months (Fig. 2a and Table 2).

**Table 2:** Changes in VAS global hand pain and FIHOA score between baseline and 6 months by treatment group

	Sinovial H-L Group (*n* = 55)			Triamcinolone Group (*n* = 45)			Difference in change scores between groups
	T0	T4	Change	T0	T4	Change	
VAS pain (mm)							
Mean ± SD	58.52 ± 16.2	29.86 ± 20.13	-28.66 ± 16.92	61.75 ± 17.56	57.15 ± 14.46	−4.25 ± 6.85	24.41***
Range	30–100	0–70	−60 − 10	30–90	30–80	-10-15	
FIHOA score							
Mean ± SD	12.16 ± 4.33	4.44 ± 4.42	−7.32 ± 5.33	12.9 ± 4.24	11.85 ± 3.53	−1.55 ± 1.3	5.77***
Range	5–25	0–16	−16-4	−5-20	5–17	−5-0	

*VAS* Visual Analogue Scale (VAS; 0–100 mm), *FIHOA* Functional Index for Hand Osteoarthritis scores (0–30 scale); T0: time of the first injection; T4: month 6

***p < 0.001 Wilcoxon rank-sum test

FIHOA score decreased in a similar manner (mean ± SD -7.32 ± 5.33 in the Sinovial H-L Group and −1.55 ± 1.3 in the Triamcinolone Group; between-group difference in the amount of change 5.77 [*p* < 0.001]). The intergroup difference in absolute levels of FIHOA at 6 months was −7.41 in favor of the Sinovial H-L Group (4.44 ± 4.42 versus 11.85 ± 3.53 in the Triamcinolone Group). The difference between groups appeared particularly evident after one month (*p* < 0.01) from the first injection and was maintained until the end of follow-up (*p* < 0.001) (Fig. 2b and Table 2).

The duration of morning stiffness was significantly reduced at 6 month follow-up in patients treated with Sinovial H-L® compared to Triamcinolone Group (mean ± SD −5.75 ± 9.45 versus −0.65 ± 2.94 min; difference of change −5.10 min [p < 0.001]) (Table 3).

**Table 3:** Changes in duration of morning stiffness, SF-36 PCS and MCS scores between baseline and 6 months by treatment group

	Sinovial H-L Group (*n* = 55)			Triamcinolone Group (*n* = 45)			Difference in change scores between groups
	T0	T4	Change	T0	T4	Change	
Duration of morning stiffness (minutes)							
Mean ± SD	9.73 ± 11.78	3.98 ± 5.21	−5.75 ± 9.45	8.85 ± 4.13	8.2 ± 3.94	−0.65 ± 2.94	−5.10***
Range	0–60	0–20	−40 − 5	0–15	0–15	-5-5	
SF-36 PCS							
Mean ± SD	35.93 ± 9.52	46.51 ± 7.28	10.57 ± 9.66	38.32 ± 8.71	39.94 ± 7.63	1.62 ± 3.12	8.95***
Range	20.37–51.41	30.98–58.81	−11.45-25.07	20.43–54.03	22.88–54.1	−3.72-8.51	
SF-36 MCS							
Mean ± SD	50.41 ± 7.68	52.23 ± 9.11	1.81 ± 10.14	41.36 ± 9.52	41.7 ± 9.36	0.34 ± 2.48	1.47*
Range	34.95–65.58	36.4–65.28	−15.84-19.55	17.21–56.32	16.13–56.09	−5.07-4.89	

*p < 0.05; ***p < 0.001 Wilcoxon rank-sum test

SF-36 PCS: Physical Components Summary, SF-36 MCS: Mental Component Summary; T0: time of the first injection; T4: month 6

**Fig. 3:**
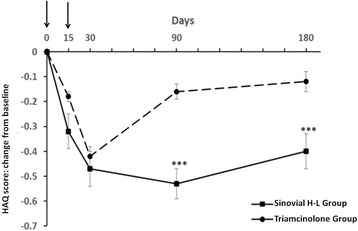
Change from baseline at each evaluation time point for the Health Assessment Questionnaire (HAQ) score in the study groups. The arrows indicate the i.a. injections. ***p < 0.001 (Wilcoxon rank-sum test) Sinovial H-L Group vs Triamcinolone Group

As showed in Fig. 3, the improvement of HAQ was more pronounced in the Sinovial H-L Group, at the 6-month visit (mean ± SD −0.4 ± 0.44 versus −0.12 ± 0.27; difference of changes −0.28 [p < 0.001]).

Finally, at the end of the study, the improvement of SF-36 PCS was significantly higher in the patients treated with HA than in those who underwent i.a steroid administration (mean ± SD 10.57 ± 9.66 versus 1.62 ± 3.12; difference of changes 8.95 [p < 0.001]), while the SF-36 MCS improvement was slightly superior (mean ± SD 1.81 ± 10.14 versus 0.34 ± 2.48; difference of changes 1.47 [*p* < 0.5]) (Table 3).

Both treatments had been well tolerated and no severe side effects were reported; only 6 patients (2 of the Sinovial H-L Group and 4 of the Triamcinolone Group) referred minor local adverse effects which had disappeared in a few days without symptomatic treatment (Table 4).

**Table 4:** Number (percentage) of patients with adverse event in the study population

	Sinovial H-L Group (n = 55)	Triamcinolone Group (n = 45)
Joint pain	2 (3.63%)	2 (4.44%)
Joint swelling	0 (0%)	1 (2.22%)
Injection site haematoma	0 (0%)	1 (2.22%)
Total number of patients with adverse event	2 (3.63%)	4 (8.88%)

## Discussion

The primary endpoint of this observational, retrospective, comparative study was to evaluate the efficacy of a hybrid formulation of HA, in comparison to triamcinolone acetonide, in reducing pain and improving hand function, after 6-months from the beginning of the i.a. treatment, in patients affected by TMJ OA.

Our results demonstrated that both i.a. therapies provided effective pain relief and joint function improvement, but the benefits achieved were statistically significantly superior in the Sinovial H-L Group than in the Triamcinolone Group during the 6-months follow-up. Furthermore, Sinovial H-L® was associated with a significant decrease in the duration of morning stiffness and with a significant improvement in HAQ and PCS-SF-36.

Despite the high prevalence of TMJ OA, in the literature there are a limited number of controlled studies comparing the efficacy of i.a. HA vs steroid and the results are often controversial [10]. The great heterogeneity of the clinical trials makes the definitive considerations difficult: indeed, the studies differ for methodology and protocol design, outcome measures, treatment (different formulations of HA and schedules) and times of follow-up.

Similar data about the efficacy of HA in pain relief and joint function improvement, in patients with TMJ OA, were reported by Stahl et al. and Fuchs et al. who observed that the effect of hyaluronate was achieved more slowly than steroid, but it was superior in the long-term [23, 24]. In 2008 Heyworth et al. evaluated, in a randomized controlled study (RCT), a course of 2 i.a. hylan, in comparison to placebo (normal saline 0.9% sodium chloride) and i.a. corticosteroid (betamethasone sodium phosphate-betamethasone acetate) in TMJ OA patients. The Authors concluded that the i.a. therapy with hylan represents a useful option for patients with TMJ OA [25]. More recently, a 6-month, single-blind, RCT was published to assess the efficacy of an i.a. formulation of HA between 500 and 1000 kDa, in comparison to betamethasone in 88 patients with TMJ OA. The Authors reported no statistically significant differences between the study groups, although a positive trend in hand function was observed in patients treated with HA. Furthermore, the efficacy of HA in functionality reached statistical significance in a subset of patients with FIHOA ≥ 5 and VAS ≥ 50 [26].

Contrasting results were reported by Bahadir et al. [27] and Mandl et al. [28] who found the superiority of i.a. steroid (triamcinolone acetonide) in comparison to HA in reducing pain and improving hand function in patients with TMJ OA.

Although the consensus statement on viscosupplementation [7] suggested to inject the TMJ under fluoroscopy or ultrasonography (US) guidance, the injections performed by an experienced physician can ensure high accuracy, as demonstrated by Mandl et al. [29].

One of the strengthes of our paper was the analysis of FIHOA, as measure of hand functionality, because of its reliability and validity in evaluating in a specific way the hand and not the arm in its globality; furthermore, this index was recently validated in Italian language and its use is according to the OMERACT Hand Osteoarthritis Group [11, 17, 30].

Furthermore, this is the first study showing a rapid efficacy of HA, similar to the effect of steroid, already evident at the end of the treatment and persistent in the follow-up period. In contrast with our results, HA was until now considered “not a rapidly acting agent”, but rather a treatment with a carryover effect on pain and function [22]. Our data may be due to several reasons, particularly consisting on the properties of the hybrid formulation of HA. This new form is featured by a bimodal MW profile, including low MW HA with preeminent anti-inflammatory and anti-apoptotic effects and high MW HA responsible for the viscoelastic properties and analgesia. The clinical benefit of the hybrid HA was recently investigated in a RCT by Papalia et al. who compared the effect of this HA to platelet-rich plasma (PRP) injections for the treatment of knee cartilage lesions among 48 professional soccer players at the end of their career [31]. International Knee Documentation Committee (IKDC), Knee injury and Osteoarthritis Outcome Summary (KOOS) and VAS were administered to the patients at baseline and after 3, 6 and 12 months. The outcome measures resulted significantly improved in both groups with a better outcome in the HA group at 3 and 6 months follow-up [31]. Furthermore, the efficacy of the hybrid association of high and low MW HA, was assessed in 20 patients affected by hip OA in comparison to a group of patients treated with high MW HA. The study demonstrated that the hybrid form provided better results both in terms of pain relief (VAS score) and improved function (Harris Hip Score) in comparison to high MW HA [32].

In the above mentioned studies the increased efficacy of the new hybrid compound was explained by the synergistic effect of different hyaluronans that can reproduce the physiological composition of synovial fluid. Indeed, the high MW component of the hybrid form restores the shock-absorbing and lubricating characteristics of depleted synovial fluid and the low MW HA stimulates the synthesis of endogenous HA and extracellular component matrix (ECM), reduces cartilage loss and chondrocytes apoptosis and decreases inflammatory activity. Furthermore, an in vitro study demonstrated that the increased ability of the hybrid form of stimulating ECM production was due to the low MW component and that the rheological properties with viscosupplements activity of the hybrid HA were the same of high MW HA [13]. Another in vitro study compared the efficacy of the hybrid formulation to that of high HA and low HA alone in cultures of human chondrocytes pre-treated with Interleukin (IL)-1β. The Authors observed that high HA, low HA and, particularly the hybrid form decreased some inflammatory biomarkers, such as Tumor Necrosis Factor (TNF)-α and IL-6, evaluated by quantitative Real Time-PCR and Bio-plex assay [33]. Furthermore, Cartilage Oligomeric Matrix Protein (COMP)-2 expression was significantly reduced when chondrocytes were treated with different hylauronans and mostly with the hybrid compound [33].

Finally, the tolerability of i.a. treatment with the new hybrid formulation of HA resulted good with only minor and transitory side effects.

However, we are aware that some limitations of our study need to be discussed. The greatest limitation lies in the fact that it is a single-center, observational, retrospective study without a randomized double-blind-placebo design. We included a relatively small number of patients not enough to perform a sub-groups analysis according to the different radiographic scores. An important source of bias in this study was the heterogeneity of the patients at basal time, in particular concerning the percentage of patients who used to smoke or with diabetes and for the cholesterol serum levels, potential confounders of treatment in OA [34, 35].

Furthermore, the present trial only compared the hybrid association of high and low MW HA to triamcinolone but not to different MW hyaluronans and the injections were performed without US guidance. Moreover, we chose a follow-up of 6 months, while previous studies suggested that a longer follow-up would be useful to understand the duration of the treatment effect [24]. For all this reasons, our results should be interpreted with caution.

## Conclusions

Despite these limitations, our results suggested that i.a. injection of the hybrid form in patients affected by TMJ OA may be more effective than triamcinolone in pain relief and joint function improvement with a rapid and persistent effect. Perhaps, the hybrid formulation seems to be a better and safe alternative treatment in comparison with triamcinolone for the management of this frequent condition. Further studies on a larger number of patients and with a longer follow-up period are needed to provide more precise therapeutic guidelines on the use of i.a. HA in patients with TMJ OA.

## Abbreviations

ACR: American College of Rheumatology
COMP: Cartilage Oligomeric Matrix Protein
ECM: Extracellular component matrix
EMA: European Medicines Agency
EULAR: European League Against Rheumatism
FIHOA: Functional Index for Hand Osteoarthritis
HA: Hyaluronic acid
HAQ: Health Assessment Questionnaire
H-L: High-Low
i.a.: intra-articular
IKDC: International Knee Documentation Committee
IL: Interleukin
KOOS: Knee injury and Osteoarthritis Outcome Summary
MCS: Mental component score
MW: Molecular weight
NHS: National Health Service
OA: Osteoarthritis
PCS: Physical component score
PRP: Platelet-rich plasma
RCT: Randomized Controlled Trial
SF: Synovial fluid
SF-36: Medical Outcomes Study 36-Item Short Form
TMJ: Trapeziometacarpal joint
TNF: Tumor Necrosis Factor
US: ultrasound
VAS: Visual Analogue Scale
